# Fabrication of electrospun cellulose-derived nanofiber membranes with enhanced stability properties of arginase

**DOI:** 10.55730/1300-0527.3424

**Published:** 2022-04-05

**Authors:** Ceyhun IŞIK, Mustafa TEKE

**Affiliations:** Department of Chemistry, Faculty of Science, Muğla Sıtkı Koçman University, Muğla, Turkey

**Keywords:** Electrospinning, nanofiber, cellulose acetate, polyvinylpyrrolidone, arginase, immobilization

## Abstract

In this study, cellulose acetate (CA)/polyvinylpyrrolidone (PVP) and CA/PVP/Mn^2+^ nanofibers were produced by the electrospinning method, and these cellulose-derived membranes were used as carriers for arginase immobilization for the first time. The structural and morphological analysis of these cellulose-derived nanofibers were determined by attenuated total reflection-Fourier to transform infrared spectroscopy (FTIR), thermogravimetric analysis (TGA), X-ray diffraction (XRD), and scanning electron microscopy (SEM). After the immobilization process, it was observed that the thermal properties of the cellulose-derived nanofibers especially improved. The optimum temperature value for free arginase was found to be 35 °C, and this value was found to be 40 °C for arginase immobilized cellulose-derived nanofibers. When the free arginase retained only about 35% of its activity at 50 °C and 60 °C after 60 min, arginase immobilized nanofibers protected 65% of their activity under the same conditions. In addition, arginase immobilized CA/PVP and CA/PVP/Mn^2+^ nanofibers managed to retain 50% of their activity even after 9 and 12 reuses, respectively.

## 1. Introduction

Enzyme immobilization is the chemical or physical attachment of soluble enzyme molecules to water-insoluble and solid carriers [1,2]. Due to the binding of enzyme molecules to suitable sites on the carrier from multiple points, the process durability, resistance to environmental changes, and mechanical properties of enzymes are improved [3,4]. Besides, immobilized enzymes have advantages such as reuse, recovery, regeneration, and easy separation from the reaction medium [5,6]. Enzyme immobilization has been one of the foci of researchers for the last few decades because of its many advantages.

Arginase (EC 3.5.3.1; L-arginine amidinohydrolase) is found in all five organism kingdoms, from unicellular prokaryotes to multicellular eukaryotes, and it is an enzyme-dependent on divalent manganese metal as a cofactor [7, 8]. It catalyzes the hydrolysis of L-arginine irreversibly to urea and L-ornithine in the last step of the urea cycle [9]. L-ornithine, which is a nonprotein amino acid and an intermediate metabolite in the urea cycle, plays a crucial role in eliminating many negative effects such as weight loss, liver diseases, strengthening immunity, and heart functions [10, 11]. L-ornithine is widely used in pharmaceutical and healthcare industries as it eliminates many adverse effects in terms of human health, and has attracted great attention in recent years due to its large market share [12, 13]. There are studies in the literature in which arginase is immobilized to different carriers for different purposes, especially for the production of L-ornithine and anticancer activity [14–16].

The properties of the carrier used in enzyme immobilization are extremely important in terms of immobilization efficiency [17]. Nanofibers of metal oxides, composite metals, and polymers have gained great importance in enzyme immobilization studies due to their unique properties [18, 19]. Although there are various methods for nanofiber production today, the electrospinning method has attracted the attention of researchers as one of the economical and simple methods in which nanofibers with homogeneous diameter, large surface area, and the porous structure can be produced [20–22]. Due to these unique properties, electrospun nanofibers are used in many fields besides biocatalysis applications [23, 24].

Polyvinylpyrrolidone (PVP) is one of the most preferred synthetic polymers in many industrial areas such as food, medicine, detergent, cosmetics, paints, plastics, and coatings due to its nontoxicity and high solubility in water and various organic solvents [25]. In addition to these properties, it has properties such as nonionic amorphous structure, adhesion strength, film formation, and surface stabilization, and therefore it is widely used in the synthesis of nanocomposites [26, 27]. Cellulose acetate (CA) is a hydrophilic, biodegradable, nontoxic, inexpensive, biocompatible, and natural biopolymer in the form of polysaccharide. Cellulose-based nanofibers are one of the interesting carrier materials for enzyme immobilization due to their easy electrospinnability, high porosity, and large surface area [28, 29].

In this study, cellulose-derived nanofibers (CA/PVP and CA/PVP/Mn^2+^) were produced by the electrospinning method to be used as a carrier in arginase immobilization. ATR-FTIR, TGA, XRD, and SEM were used to determine the morphological and structural properties of these cellulose-derived nanofibers. In order to improve the stability properties of the arginase enzyme such as optimum pH, optimum temperature, thermal stability, and reusability, arginase was immobilized by arginase adsorption and crosslinker method on these cellulose-derived nanofibers.

## 2. Experimental

### 2.1. Materials

Cellulose acetate (M_w_ = 50,000 Da), polyvinylpyrrolidone (M_w_ = 1,300,000 Da), L-arginase (from bovine liver, 2.5 KU), L-ornithine, α-Isonitrosopropiophenone (ISPF), manganese (II) chloride (MnCl_2_), acetic acid, Coomassie Brillant Blue G-250, ethanol, glutaraldehyde (GA), manganese (II) acetate tetrahydrate (Mn(CH_3_COO)_2_ 4H_2_O) and all other chemicals were purchased from Sigma-Aldrich.

### 2.2. Preparation of cellulose-derived nanofibers with electrospinning

Different concentrations of CA/PVP polymer solutions (7.5%, 10% and 12.5% for CA, 2.5% and 5% for PVP) were prepared from acetic acid/ethanol mixture. Firstly, CA was completely dissolved in acetic acid and PVP was completely dissolved in ethanol. Then, these prepared solutions were mixed in equal volumes until a homogeneous solution was obtained. All these processes were carried out at room temperature. The polymer solution was put into the syringe. The syringe pump (New Era Pump Systems Inc.) was integrated into the electrospun system (Inovenso nanospinner). Voltage (13, 15, 17 kV), needle tip-collector distance (15, 17, 19 cm) and flow rate (0.3, 0.4, 0.6 mL/h) were studied as optimization parameters of electrospun process. In order to prepare CA/PVP/Mn^2+^ nanofiber, the concentrations of CA (12.5%) and PVP (5%) were kept constant and polymer solution was prepared with different Mn^2+^ concentrations (0.1, 0.2, 0.3%). Firstly, Mn(CH_3_COO)_2_ 4H_2_O was completely dissolved in ethanol and then PVP was added. This mixture was then mixed with an equal volume of CA solution. This prepared solution was put into a syringe and integrated into the electrospun system to produce nanofibers. As with the CA/PVP nanofiber, optimization parameters of an electrospun process such as voltage (13, 15, 17 kV), needle tip-collector distance (13, 15, 17, 19 cm), and flow rate (0.3, 0.5 mL/h) were determined for the production of this nanofiber.

### 2.3. Characterization of cellulose-derived nanofibers

SEM (JEOL JSM 7600F) was used to observe the changes in the surface morphology of the nanofibers before and after the immobilization process. In addition, the surface morphologies of the nanofibers were also examined after reuse. The nanofiber samples were sputter-coated with gold for a min at 15 mA before analysis. Surface groups and the chemical structure of nanofibers were determined by Fourier Transform Infrared Spectroscopy (Thermo Scientific Nicolet iS-5ATR/FTIR Spectrometer, FTIR). Crude polymers and nanofibers were analyzed in the range of 4000–500 cm^−1^ with a wave number accuracy of 0.01 cm^−1^. X-Ray Diffraction (XRD) was determined using the Rigaku SmartLab model diffractometer under a wide-angle (10–60º) scanning range with a step size of 0.01º and scanning mode of 0.3, (Cu target of 1.5412 Å). The current and voltage were 30 mA and 40 kV, respectively. Thermal analysis of both the raw polymer and cellulose-derived nanofibers was analyzed using the Perkin Elmer Thermal Gravimetric Analyzer (TGA) 4000. The samples were taken 5–10 mg and they were analyzed from 50 °C to 650 °C, at a heating rate of 10 °C/min in N_2_ atmosphere.

### 2.4. Immobilization of arginase onto cellulose-derived nanofibers

Nanofibers were used as carriers for arginase immobilization. Adsorption and cross-linking methods were used in the immobilization process. To determine the immobilization parameters, arginase concentration (0.25; 0.5; 1.0; 2.0; 4.0 U/mL), nanofiber amount (2.5; 5.0; 7.5; 10.0; 12.5; 15.0 mg), adsorption time (5; 10; 15; 20; 25; 30 min) and GA amount (1; 2; 3; 4; 5; 6%) were examined. In order to immobilize the enzyme on the nanofibers by adsorption method, a known number of nanofibers was taken and mixed with 1 mL of arginase solution for a known time. Then, a known amount of GA was added to this mixture and mixed for the known amount of time to crosslink the enzyme to the nanofiber. Finally, the solution in the mixture was removed and washed several times with TBS buffer to remove excess GA and unbound enzyme molecules on the nanofiber.

### 2.5. Determination of amount of protein

The amount of protein in the reaction medium before and after immobilization was determined by using Bradford method [30].

### 2.6. Arginase activity assay

A colorimetric method based on measuring the amount of urea formed as a result of hydrolysis of L-arginine was used to determine arginase activity [31]. The enzyme solution was prepared using TBS buffer (50 mM, pH 7.5 Tris/HCI, 0.1% bovine serum albumin, and containing 0.1 M NaCl). Arginine (400 μL) (50 mM, pH 9.7), 600 μL of 10 mM MnCl_2_ (prepared with 50 mM, pH 7.5 Tris-HCl solution), and 200 μL of arginase solution were placed in the test tube and incubated at 37 °C for 1 h. The reaction was stopped by adding 2400 μL of acid solution (H_2_SO_4_/H_3_PO_4_/H_2_O (1:3:7)). Then, 200 μL of 5% ISPF prepared with ethanol was added to the mixture and incubated in boiling water for 45 min. Finally, the test tube was kept in the dark for 10 min since the reaction between ISPF and urea was light-sensitive, and the arginase activity was determined spectrophotometrically at 540 nm [32]. Hydrolysis of 1 μmol L-arginine to urea in 1 min at 37 °C and pH 9.5 was defined as 1 U of arginase activity.

### 2.7. Characterization of arginase immobilized cellulose-derived nanofibers

In order to determine the optimum temperature values of free arginase and arginase immobilized cellulose-derived nanofibers, arginase activity was determined by changing the temperature between 20 **°C** and 70 **°C**. In order to determine the thermal stability properties of free arginase and arginase immobilized cellulose-derived nanofibers, both free arginase and arginase immobilized electrospun nanofibers were kept at temperatures ranging from 4 **°C** to 80 **°C** for 60 min, and then the activity was determined.

In order to determine the optimum pH values of free enzyme and arginase immobilized cellulose-derived nanofibers, activity measurements were made using 50 mM buffer solutions with pH varying between 3 and 12. To determine pH stability properties, free enzyme and arginase immobilized cellulose-derived nanofibers were kept in 50 mM buffer solution with pH between 3 and 11 for 1 h, then activity measurement was performed.

To determine the reusability of arginase immobilized cellulose-derived nanofibers, the activity measurement was repeated until the initial activity value decreased by 50%. After each activity assay, arginase-immobilized nanofibers were washed three times with TBS buffer and fresh arginine solution was added to the medium before each activity assay.

The arginase activity assay was studied for different concentrations (5–50 mM) of arginine solution under activity assay conditions to determine the Michaelis-Menten constant (K_m_) and the maximum velocity (V_max_) of both free arginase and arginase immobilized cellulose-derived nanofibers.

## 3. Results and discussion

### 3.1. Determination of operational parameters of cellulose-derived nanofibers

Injection speed, the distance between the needle and collector, electrical voltage, CA, PVP, and Mn^2+^ concentration were determined as optimization parameters for electrospun nanofibers. Operational parameters and observation results for CA/PVP and CA/PVP/Mn^2+^ nanofibers are given in Table. Injection speed (0.4 mL/h), 15 cm needle-collector distance, 15 kV electrical voltage, 12.5% CA, and 5% PVP concentration were found for CA/PVP nanofiber. The optimum parameters for CA/PVP/Mn^2+^ nanofiber were found to be 0.6 mL/h injection speed, 15 cm needle-collector distance, 17 kV electrical voltage, 12.5% CA, 5% PVP, and 0.1% Mn^2+^ concentration. When the optimal parameters of CA/PVP and CA/PVP/Mn^2+^ nanofibers were compared, higher electrical voltage and higher injection speed were used for the production of CA/PVP/Mn^2+^ nanofiber. The concentrations of polymers in both electrospun nanofiber structures were the same, but in CA/PVP/Mn^2+^ nanofiber, Mn(CH_3_COO)_2_ 4H_2_O was added to the structure as an Mn^2+^ source, unlike the CA/PVP nanofiber. In order to produce nanofibers by the electrospinning method, the intensity of the applied voltage must be greater than the surface tension of the polymer solution drop at the syringe tip. Because of the Mn(CH_3_COO)_2_ 4H_2_O added to the polymer solution, the density of manganese and acetate ions in the solvent and the force between the solvent molecules increased, so the surface tension of the polymer solution increased. For this reason, a higher injection speed rate and electrical voltage were applied when producing the CA/PVP/Mn^2+^ nanofiber.

### 3.2. Characterization of cellulose-derived nanofibers

**Figure 1:**
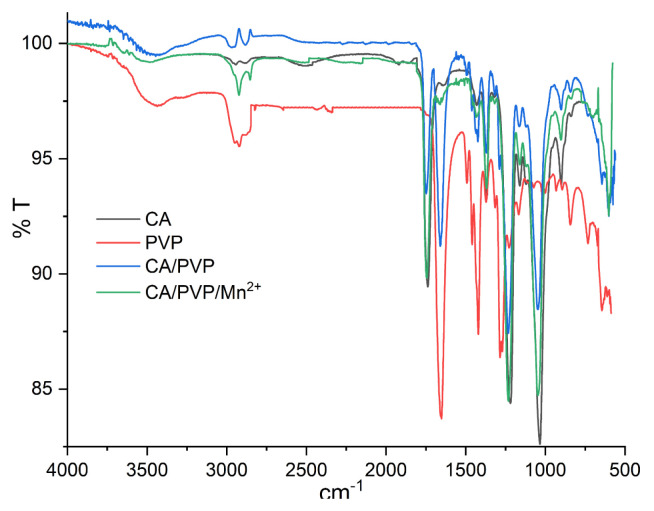
ATR-FTIR spectra of CA, PVP, CA/PVP and CA/PVP/Mn^2+^ nanofibers.

ATR-FTIR spectrums of crude CA, crude PVP, and cellulose-derived nanofibers were given in Figure 1. In the spectrum of CA, the bands around 3486 cm^−1^ and 1740 cm^−1^ indicated the presence of −OH and −COOH groups, respectively. The band at 1368 cm^−1^ was due to bending vibrations resulting from −CH_3_ deformation in the acetate substituent groups. The bands at 1220 cm^−1^ and 1035 cm^−1^ were shown C-O-C vibration stretching and C-O stretching, respectively. In the spectrum of PVP, the absorption band at 1285 cm^−1^ indicated C-N bending vibration caused by the pyrrolidone structure. The bands around 1420 cm^−1^ and 1375 cm^−1^ were shown the CH deformation forms from the CH_2_ group. Absorption bands caused by the stretching vibration of C=O in the pyrrolidone group were seen at 1654 cm^−1^. Ternary CH (2853 cm^−1^), symmetric CH_2_ stretching (chain: 2921 cm^−1^; ring: 2883 cm^−1^), and symmetric CH_2_ stretching (chain: 2983 cm^−1^, ring: 2954 cm^−1^) bands were also observed due to CH extension modes. In addition, since PVP is a bisubstituted amide, absorption bands of amines around 3400–3500 cm were not observed. When the spectrum of the CA/PVP nanofiber was examined, it was seen that the peaks of the raw CA and raw PVP were preserved. A slight shift in peaks was observed due to the tendency of the carbonyl group of PVP (acting as proton acceptor) and the hydroxyl group of CA (acting as proton donor) to form hydrogen bonds. When the spectrum of CA/PVP/Mn^2+^ nanofiber and the spectrum of CA/PVP nanofiber were compared, significant changes were observed in the characteristic bands showing the presence of C=O with PVP at 1654 cm^−1^. In addition, changes were observed in the characteristic bands of CA, which are around 3486 cm^−1^ and 1740 cm^−1^, indicating the presence of −OH and −COOH groups, respectively. It can be said that Mn^2+^ ions interact with both the carbonyl groups of PVP and the hydroxyl groups of CA via coordination.

**Figure 2:**
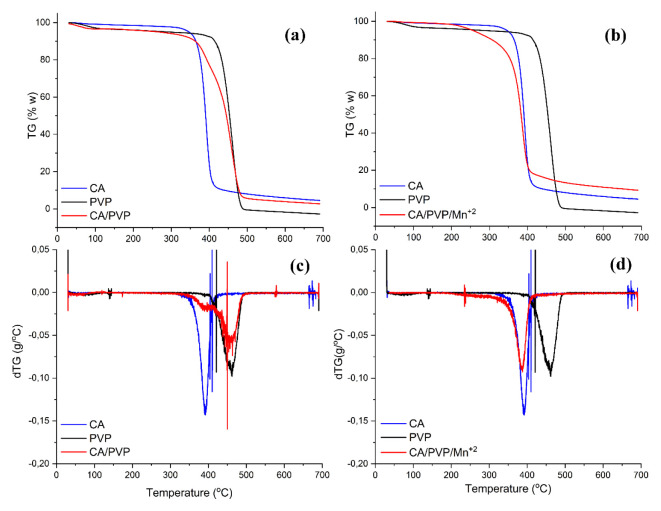
TG (a), (b) and DTG (c), (d) curves of CA, PVP and cellulose-derived nanofibers.

Thermogravimetric (TGA) analysis of crude CA, crude PVP, and cellulose-derived nanofibers are given in Figures 2a, 2b, 2c, and 2d. Decomposition of the polymer for the crude CA structure occurred in three steps. In the first step, a 4% reduction in mass was observed for the raw CA structure below 330 °C. The reason for this decrease may be the removal of volatile substances and/or absorbed moisture in the structure. In the second stage, where the main degradation was observed, approximately 90% of the initial mass was removed from the structure between the temperatures of 330–430 °C. It was defined that the reduction in mass was due to the main thermal degradation reactions of the cellulose acetate chains in the literature [33]. At the end of the third stage, which took place at temperatures between 430–700 °C, it was observed that approximately 96% of the initial mass was removed from the structure. At the end of the process, the ash residue amount of the building was found to be 4%. This three-step degradation curve of CA corresponds to the steps proposed by Chatterjee (1968) for the thermal degradation of cellulose-based materials [34]. The visible peak was observed at 390 °C which represents the maximum degradation temperature of CA in the dTG curve of CA.

For crude PVP, three-step degradation was observed. In the first step, a 3% reduction in mass was observed in the raw PVP structure below 95 °C. This decrease may be due to the moisture content in the structure. In the second stage, approximately 8% of the initial mass was removed from the structure between the temperatures of 95–390 °C. At this stage, it was reported that mass loss occurred due to the access of oligomers, low molecular weight substances, moisture, and residual solvent in the structure with the increase in temperature [35]. In the last step, which took place at temperatures between 390–488 °C, all of the initial mass was removed and the structural polymer was completely degraded. The maximum degradation temperature of PVP was found to be 460 °C.

As seen in TG and dTG curves of CA/PVP and CA/PVP/Mn^2+^ nanofibers, it was observed that the degradation curves of the nanofibers were different from the degradation curves of the crude polymers. These differences may be due to differences in textural structures between polymers, interactions between polymers and possible multiple noncovalent interactions of Mn^2+^ ions with the polymers. For CA/PVP nanofiber, a decrease of approximately 3% in mass was observed due to the evaporation of water from the structure at 83 °C. It was observed that approximately 7.5% of the initial mass was removed from the structure between 95–340 °C. This decrease may be due to the removal of low-weight substances/oligomers/moisture/solvent from the structure. The final stage took place between 340–485 °C and about 93% of the mass was removed. The reduction in mass at this stage was smoother compared to the crude polymers. This may be due to hydrogen bonds formed between −OH, C=O and −NH groups. At the end of the analysis, the ash residue amount for the CA/PVP nanofiber structure was found to be 3%. The maximum degradation temperature for CA/PVP nanofiber was determined as 455 °C. It can be said that CA/PVP nanofiber has higher thermal stability than raw CA and raw PVP, and this is due to possible multiple noncovalent interactions between CA and PVP in the structure of the nanofiber.

For CA/PVP/Mn^2+^ nanofiber structure, 2% decrease in mass was observed below 200 °C due to the removal of water from the structure. In the second stage at 200–410 °C, about 81% of the initial nanofiber mass was decreased. In the final stage, no significant mass loss was observed between 410–700 °C. This may be due to the fact that the PVP in the structure completely decomposes at 488 °C and only metal ion residues remain in the structure. The ash residue amount was found to be 9% at the end of the analysis. Due to the manganese metal in the structure of the CA/PVP/Mn^2+^ nanofiber, the amount of ash residue was found to be higher than the CA/PVP nanofiber. The maximum degradation temperature of the CA/PVP/Mn^2+^ nanofiber membrane structure was determined as 390 °C in the dTG curve. The degradation temperature of the CA/PVP nanofiber structure was found to be higher than the CA/PVP/Mn^2+^ nanofiber structure. This may be due to the fact that the Mn^2+^ ions in the structure acted as a catalyst in the degradation of the structure.

**Figure 3:**
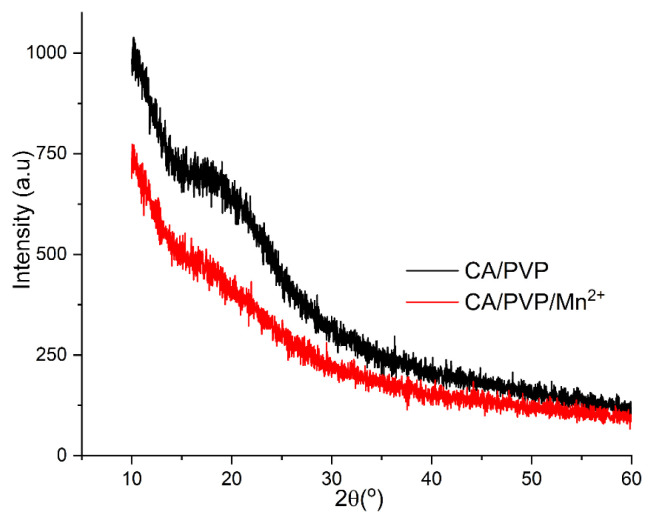
Wide-angle XRD patterns of cellulose-derived nanofibers.

Wide-angle XRD patterns of CA/PVP and CA/PVP/Mn^2+^ nanofibers are given in Figure 3. A shoulder nearly at 2θ = 19.6° was observed for both CA/PVP and CA/PVP/Mn^2+^ nanofibers. It can be said that both nanofibers are in amorphous structure and not in regular molecular structure. Also, as seen in Figure 3, the intensity of the spectrum of the CA/PVP/Mn^2+^ nanofiber was less than the intensity of the spectrum of the CA/PVP nanofiber. It can be said that the reason for this situation is due to the blending of manganese ions in the structure of the CA/PVP/Mn^2+^ nanofiber with the CA and PVP polymers at the molecular level. Similar studies in which the peak intensity decreases with the addition of another polymer or metal ion to the structure are available in the literature [36].

**Figure 4:**
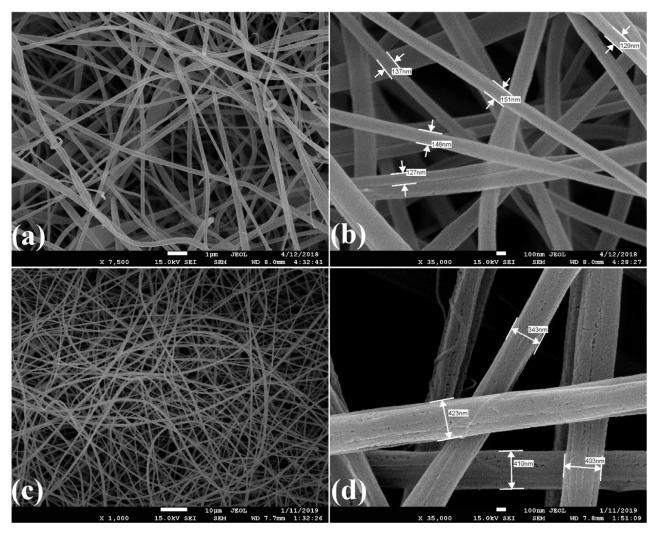
SEM images of (a), (b) CA/PVP nanofibers (c), (d) CA/PVP/Mn^2+^ nanofibers.

SEM images of CA/PVP and CA/PVP/Mn^2+^ nanofibers are shown in Figures 4a, 4b, Figure 4c, and 4d, respectively. As seen in Figures 4a and 4b, the fibers were randomly positioned, smooth, beadless and fibers had almost the same diameter. The diameters of CA/PVP nanofibers were found to be around 121–151 nm. As seen in Figures 4c and 4d, CA/PVP/Mn^2+^ nanofiber was observed that the fibers were randomly positioned, slightly rough, rounded, no bead formation was observed, and their average diameter was around 343–423 nm. It was seen that the diameter of CA/PVP/Mn^2+^ nanofibers was higher than the diameter of CA/PVP nanofibers. This can be interpreted as the addition of manganese ions to the polymer solution causing an increase in the viscosity of the solution and as a result, the nanofiber diameters increase. There is a similar study in the literature in which nanofibers with larger fiber diameters are formed as a result of increasing the viscosity of the polymer solution [37].

### 3.3. Optimization and characterization of arginase immobilization on cellulose-derived nanofibers

**Figure 5:**
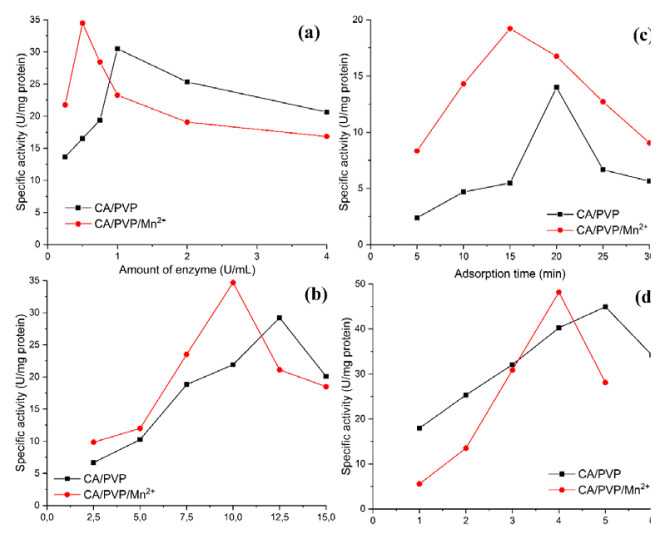
Optimization parameters results of arginase immobilized cellulose-derived nanofibers.

Adsorption and cross-linking methods were used for the immobilization of arginase on cellulose-derived nanofibers. The enzyme amount, nanofiber amount, adsorption time, and crosslinker concentration were determined in the optimization studies of the immobilization process. Optimization parameters results for arginase immobilized cellulose-derived nanofibers are presented in Figure 5.

As seen in Figure 5a, the optimum enzyme amount was found to be 1 U/mL for CA/PVP nanofiber and 0.5 U/mL for CA/PVP/Mn^2+^ nanofiber (10 mg nanofiber, 20 min adsorption time, and 3% GA). The specific activity for both nanofibers gradually decreased at amounts above the optimum enzyme amount. One of the most important reasons for this may be that the amount of the carrier remains constant despite the increase in the amount of enzyme in the reaction medium. Compared to CA/PVP nanofiber, the possible reason for lower optimum enzyme amount and higher enzymatic activity of CA/PVP/Mn^2+^ nanofiber may be due to the presence of manganese ion in the structure of CA/PVP/Mn^2+^ nanofiber.

The arginase immobilized CA/PVP and CA/PVP/Mn^2+^ nanofibers showed the optimum activity when the values of nanofiber amount were 12.5 and 10 mg, respectively (Figure 5b). Probably, at values below the optimum amount of nanofiber, there were not enough carriers to bind the enzyme molecules. At values above the optimum amount of carrier, the carrier may have sterically inhibited the interaction that should occur between the immobilized enzyme and the substrate.

The surface of CA/PVP and CA/PVP/Mn^2+^ nanofibers reached saturation at 20 min and 15 min, respectively (Figure 5c). After these times, the immobilized enzyme molecules may have started to desorb from the carrier surface. In addition, it can be said that the manganese ions in the CA/PVP/Mn^2+^ structure contribute to the saturation of these nanofibers in a shorter time by the enzyme.

In order to determine the optimum glutaraldehyde concentration, the amount of enzyme, carrier amount, and adsorption time were kept constant and immobilization was performed at glutaraldehyde concentrations ranging from 1% to 6%. As seen in Figure 5d, the optimum amount of glutaraldehyde was found to be 5% for CA/PVP nanofiber and 4% for CA/PVP/Mn^2+^ nanofiber. At values below the optimum GA amount, the amount of GA in the environment may have been insufficient for the immobilization of enzyme molecules to nanofibers. The active center of the arginase enzyme was affected due to some conformational changes at higher values than the optimum GA concentration.

### 3.4. Temperature properties

**Figure 6:**
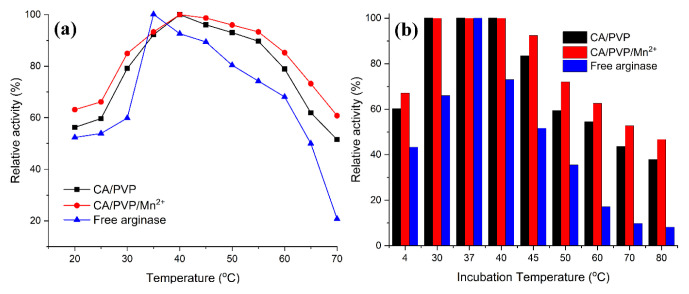
(a) The optimum temperature profiles and (b) the thermal stabilities of free arginase and arginase immobilized cellulose-derived nanofibers.

In order to determine the temperature profiles of arginase immobilized cellulose-derived nanofibers and free arginase, activity determination was performed at temperature ranges from 20 to 70 °C (Figure 6a). The optimum temperature for the free arginase was 35 °C. After immobilization, the optimum temperature value changed and it was found 40 °C for both cellulose-derived nanofibers. Furthermore, at 70 °C, the free enzyme retained only 20% of its activity, while the arginase immobilized CA/PVP and CA/PVP/Mn^2+^ nanofibers protected 52% and 61% of their activity, respectively. There is a similar study in which an increase in the optimum temperature value was observed after enzyme immobilization. Dala and Szajani reported that arginase (from the bovine liver) was immobilized on polyacrylamide support and the optimum temperature for free arginase was 40 °C and after immobilization, this value changed and it was 60 °C [15].

Free arginase and arginase immobilized cellulose-derived nanofibers were incubated at different temperatures (4–80 °C) for 1 h. Then, an activity assay was performed to determine the thermal stability properties. The results were presented in Figure 6b. Arginase immobilized cellulose-derived nanofibers showed higher activity than free arginase enzyme at all temperatures. Especially at 30, 37, and 40 °C, cellulose-derived nanofibers did not show any loss of their activity. At 60 °C, when arginase immobilized CA/PVP/Mn^2+^ and CA/PVP nanofibers kept 63% and 54% of their activity respectively free arginase lost 83% of its activity. It can be said that arginase immobilized CA/PVP/Mn^2+^ nanofiber membranes are thermally more stable than arginase immobilized CA/PVP nanofiber membranes. The increase in stability observed after the immobilization process may also be due to the high energy required to break the stable bonds formed between the nanofiber and the enzyme. In addition, nanofibers may prevent the enzyme molecules from being affected by the intensity of the heat, especially at high temperatures, causing the enzyme not to be denatured. Dala and Szajani reported that arginase immobilized polyacrylamide support protected its all activity at 40 °C after 120 min, it lost 80% of its activity at 60 °C after 60 min, and it lost the whole of its activity after 30 min at 70 °C [15]. In another study, Zhang et al. immobilized arginase on chitosan particles and it maintained only 40% of its activity at 60 °C after 60 min [38].

### 3.5. pH properties

**Figure 7:**
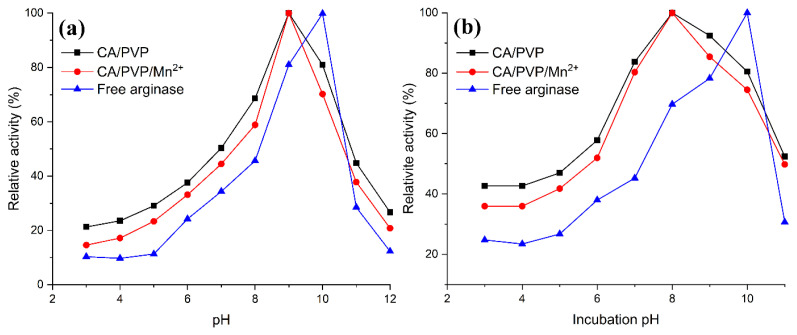
(a) The optimum pH profiles and (b) the pH stabilities of free arginase and arginase immobilized cellulose-derived nanofibers.

After immobilization of the enzyme to the carrier, the effect of pH on enzymatic activity may vary depending on the nature of the carrier. As shown in Figure 7a the optimum pH of the free arginase was 10.0. After immobilization, the optimum pH value for both cellulose-derived nanofibers changed and was found to be pH 9.0. Especially in acidic conditions, cellulose-derived nanofibers showed higher activity than free arginase. The shift to the weakly basic region at optimum pH and higher activity in acidic conditions can be explained by the fact that CA, which has negatively charged surfaces in nanofiber structures, protects the microenvironment of the enzyme in acidic conditions. Unissa et al. reported that the optimum pH value did not alter after human arginase I immobilization on silver nanoparticles [14]. In the report of Dala and Szajani, arginase was immobilized on polyacrylamide support, and the optimum pH for the free arginase was 11.0 and after immobilization, this value was 9.5 [15].

In order to determine the pH stability of free arginase and arginase immobilized cellulose-derived nanofibers, the immobilized nanofibers were incubated for 1 h in buffer solutions with pH between 3 and 11. As given in Figure 7b, it was observed that cellulose-derived nanofibers had higher pH stability than free enzymes under acidic conditions. In addition, arginase immobilized CA/PVP nanofibers showed higher pH stability than CA/PVP/Mn^2+^ nanofibers at the same pH conditions. This can be explained by the chelation of manganese ions in the CA/PVP/Mn^2+^ nanofiber with groups that can act as buffers against pH changes in the CA and PVP structure.

### 3.6. Kinetic parameters

After the immobilization process, changes in the kinetic behavior of the enzyme can be observed due to changes in the three-dimensional structure of the enzyme, steric effects, changes in the microenvironment of the enzyme, and diffusion effects. In order to observe the changes in kinetic behavior after arginase immobilization, activity determination was performed using L-arginine in the range of 5–50 mM. The K_m_ and V_max_ values of free arginase, arginase immobilized CA/PVP, and CA/PVP/Mn^2+^ nanofibers were found as 1.06, 1.19, 1.18 mM, and 50.25, 37.74, 41.32 U/mg protein, respectively. A small increase in K_m_ values of both immobilized nanofibers was observed when compared with the free enzyme. It can be said that this increase in K_m_ value negatively affects the conformation of arginase after the immobilization process of the functional groups on the carrier and the affinity of the enzyme to the substrate decreases. It can be interpreted that the decrease in V_max_ value is due to the fact that the substrate reaches the active site of the immobilized enzyme more difficult due to the changes in the conformational structure of the enzyme after immobilization.

### 3.7. Reusability

**Figure 8:**
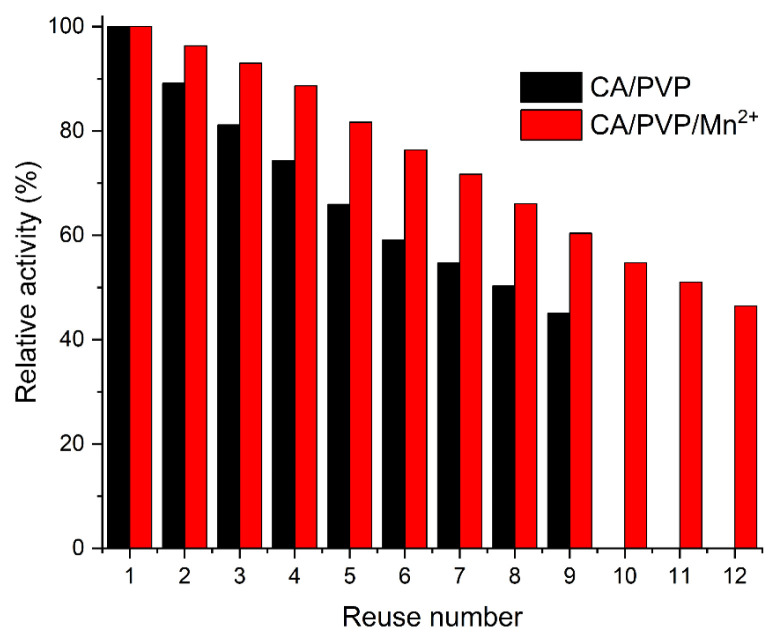
The reusability of arginase immobilized cellulose-derived nanofibers.

One of the most important advantages of the immobilization process is reusability. The number of reused arginase immobilized cellulose-derived nanofibers was determined by measuring arginase activity repeatedly. As seen in Figure 8, the activities of arginase immobilized CA/PVP and CA/PVP/Mn^2+^ nanofibers decreased to less than 50% after 9 and 12 reuses, respectively. According to this result, it can be said that manganese ions in the CA/PVP/Mn^2+^ structure provide additional stability to the enzyme after immobilization.

**Figure 9:**
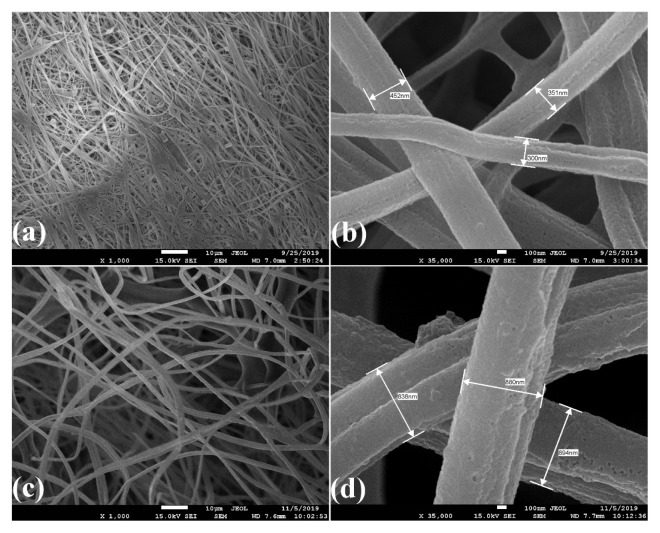
SEM images of (a), (b) arginase immobilized CA/PVP nanofiber; (c), (d) arginase immobilized CA/PVP nanofiber after 9 reuses.

SEM images of arginase immobilized CA/PVP nanofibers are shown in Figures 9a and 9b. Compared with the SEM images of the CA/PVP nanofibers given in Figure 4a, it was seen that the nanofibers could not preserve their structure after immobilization and their average diameter increased to around 300–452 nm. It has been reported in the literature that the deterioration in the nanofiber structure after the immobilization process is caused by the crosslinker used to immobilize the enzyme molecules to the carrier [39,40]. It was observed that the nanofiber structure deteriorated, their surfaces were rough and their diameters increased after 9 reuses (Figure 9c). Since the changes in the structures of the nanofibers after reuse cause denaturation or desorption of the immobilized enzyme, a decrease in the efficiency of the immobilized arginase enzyme may have been observed.

**Figure 10:**
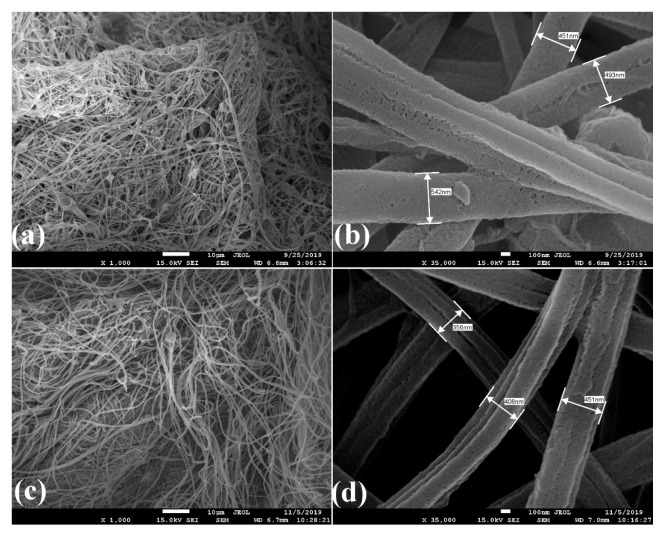
SEM images of (a), (b) arginase immobilized CA/PVP/Mn^2+^ nanofiber; (c), (d) arginase immobilized CA/PVP/Mn^2+^ nanofiber after 12 reuses.

SEM images of arginase immobilized CA/PVP/Mn^2+^ nanofibers are given in Figures 10a and 10b. When the CA/PVP/Mn^2+^ nanofibers given in Figure 4c were compared with Figures 10a and 10b, it was seen that the nanofibers partially preserved their structure after immobilization, their structures did not change and their average diameter was around 452–542 nm. There are similar studies in the literature in which the structures of nanofibers are preserved after the immobilization process [41]. After 12 reuses, it was observed that the structure of arginase immobilized CA/PVP/Mn^2+^ nanofibers deteriorated, the fiber diameters were reduced to around 356–451 nm and they were porous (Figures 10c and 10d).

## 4. Conclusions

In this study, CA/PVP and CA/PVP/Mn^2+^ nanofibers were produced by the electrospinning method and arginase was successfully immobilized on these cellulose-derived nanofibers by adsorption and cross-linking methods. At 70 °C, free arginase showed only 20% of its activity, while arginase immobilized cellulose-derived nanofibers retained more than 50% of their activity. When free enzyme showed 17% activity, immobilized CA/PVP and CA/PVP/Mn^2+^ nanofibers kept 63% and 54% of their activity, respectively at 60 °C after 60 min. Arginase immobilized CA/PVP and CA/PVP/Mn^2+^ nanofibers retained more than 50% of their activity even after 8 and 11 reuses, respectively. Especially manganese-doped cellulose-derived nanofiber showed more stability than CA/PVP nanofiber in terms of reuse and thermal stability. This can be interpreted as manganese ions providing additional stability to the nanofiber structure. In addition, a shift to the acidic region was observed at the optimum pH value after immobilization. As a result, it can be said that a new, alternative and biocatalytic method is presented with cellulose-derived nanofiber membranes for the production of L-ornithine, which is widely used in the healthcare industry.

## Figures and Tables

**Table:** Operational parameters and observation of cellulose-derived nanofibers.

CA concentration (%)	PVP concentration (%)	Mn^2+^ concentration (%)	Voltage (kV)	Needle-collector distance (cm)	Injection speed (mL/h)	Observation*
10	2.5	−	15	16	0.3	−
10	2.5	−	16	18	0.4	+
10	5	−	15	15	0.4	+
10	5	−	16	17	0.4	+
12.5	2.5	−	15	20	0.3	+
12.5	2.5	−	17	18	0.4	++
12.5	5	−	15	17	0.5	++
**12.5**	**5**	−	**15**	**15**	**0.4**	**+++**
12.5	5	−	16	18	0.4	++
12.5	5	0.1	15	15	0.3	−
12.5	5	0.1	17	15	0.5	++
**12.5**	**5**	**0.1**	**17**	**15**	**0.6**	**+++**
12.5	5	0.2	17	15	0.6	−
12.5	5	0.2	19	17	0.5	++
12.5	5	0.2	19	20	0.6	+
12.5	5	0.3	17	15	0.5	+
12.5	5	0.3	19	13	0.5	++
12.5	5	0.3	20	15	0.6	+

* Criteria of positive (+) observation: The Taylor cone formation during electrospinning; fiber formation, no formation of polymer droplets at the needle tip or on the collector; stable system; easy removal of fiber from the collector; mechanically stable fiber.
